# Optimizing *E. coli* as a formatotrophic platform for bioproduction *via* the reductive glycine pathway

**DOI:** 10.3389/fbioe.2023.1091899

**Published:** 2023-01-16

**Authors:** Seohyoung Kim, Néstor Giraldo, Vittorio Rainaldi, Fabian Machens, Florent Collas, Armin Kubis, Frank Kensy, Arren Bar-Even, Steffen N. Lindner

**Affiliations:** ^1^ Max Planck Institute of Molecular Plant Physiology, Potsdam, Germany; ^2^ b.fab GmbH, Köln, Germany; ^3^ Department of Biochemistry, Charité—Universitätsmedizin Berlin, Corporate Member of Freie Universität Berlin and Humboldt-Universität zu Berlin, Berlin, Germany

**Keywords:** formate (compound CID: 283), *E. coli—Escherichia coli*, C1-assimilation, bioeconomy and circular economy, bioproduction, synthetic biology (synbio), metabolic rewiring

## Abstract

Microbial C1 fixation has a vast potential to support a sustainable circular economy. Hence, several biotechnologically important microorganisms have been recently engineered for fixing C1 substrates. However, reports about C1-based bioproduction with these organisms are scarce. Here, we describe the optimization of a previously engineered formatotrophic *Escherichia coli* strain. Short-term adaptive laboratory evolution enhanced biomass yield and accelerated growth of formatotrophic *E. coli* to 3.3 g-CDW/mol-formate and 6 h doubling time, respectively. Genome sequence analysis revealed that manipulation of acetate metabolism is the reason for better growth performance, verified by subsequent reverse engineering of the parental *E. coli* strain. Moreover, the improved strain is capable of growing to an OD_600_ of 22 in bioreactor fed-batch experiments, highlighting its potential use for industrial bioprocesses. Finally, demonstrating the strain’s potential to support a sustainable, formate-based bioeconomy, lactate production from formate was engineered. The optimized strain generated 1.2 mM lactate —10% of the theoretical maximum— providing the first proof-of-concept application of the reductive glycine pathway for bioproduction.

## Introduction

The valorization of carbon dioxide is a major challenge in our society and is subject to intense research and investment. Naturally, the biological transformation of carbon dioxide takes place in plants and algae. However, photosynthesis greatly suffers from low-energy conversion efficiencies in the range of 3%–5% (Janssen et al., 2003; Zhu et al., 2010). Pure chemical transformation, where various chemicals such as urea, methanol, and salicylic acid can be derived directly from carbon dioxide, might be another option (He et al., 2013; Wong, 2014). However, the processes require extreme conditions and their product spectrum and product selectivity are limited. A promising solution is to combine the individual strengths of biological and chemical processes to circumvent their weaknesses. In such an approach, carbon dioxide can be reduced electrochemically, using renewable energy sources, to various C1 compounds. Among them is formic acid, which can be produced with a very high faradaic efficiency and can be utilized as a feedstock for microorganisms (Li et al., 2012; Liu et al., 2016; Yishai et al., 2016). Formic acid is one of the most suitable C1 compounds for the bioindustry, especially because of its solubility and low toxicity (Claassens et al., 2019).

**FIGURE 1:**
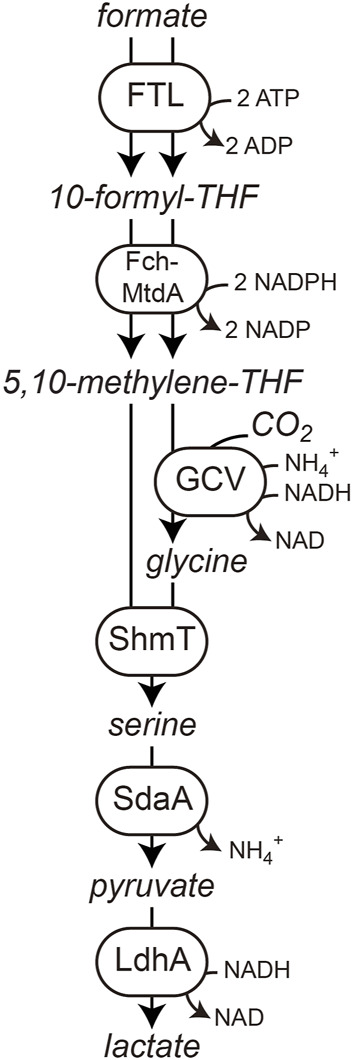
Reductive glycine pathway as operating in the formatotrophic *E. coli* strain. Displayed is formate and CO_2_ conversion to lactate as a final bioproduct.

However, engineering natural C1-assimilating microorganisms to produce value-added biochemicals from single-carbon compounds is often limited by their poor growth characteristics and recalcitrance to genetic modification. Various natural C1 assimilation routes have been identified, including the reductive pentose phosphate cycle, the reductive acetyl-CoA pathway, and the reductive citric acid cycle from various domains of life (Berg et al., 2010; Bar-Even et al., 2012a; Bar-Even et al., 2012b). Nature has optimized these microorganisms, enzymes, and metabolic fluxes over billions of years, rendering attempts to improve energy consumption and carbon-fixation efficiency very challenging. Implementing natural or new-to-nature synthetic pathways for C1 assimilation into biotechnologically important microbes such as *E. coli*, which do not naturally grow on C1 compounds, can solve these problems. Assimilation of C1 compounds such as CO_2_, formate, CO, or methanol *via* their respective assimilation pathways has been receiving increased attention (Schwander et al., 2016; Meyer et al., 2018; Gleizer et al., 2019; Bang et al., 2020; Chen et al., 2020; Gassler et al., 2020). Besides the reductive acetyl-CoA pathway, the synthetic and oxygen-tolerant reductive glycine (rGly) pathway is the most efficient formate assimilation pathway (Bar-Even et al., 2013; Claassens et al., 2022). This pathway was recently successfully engineered in *E. coli* for generation of all biomass from formate and CO_2_ (Figure 1), reaching a doubling time of 9 h and a biomass yield of 2.3 g cell dry weight/mol (CDW/mol) formate (Kim et al., 2020). However, so far bioproduction *via* synthetic C1-assimilation pathways has not been reported.

In this work, growth performance of the formatotrophic *E. coli* strain was improved using an adaptive laboratory evolution approach on formate under 10% CO_2_ atmosphere. Genome sequencing identified mutations that apparently increased the growth performance on formate, and their effects were verified through reverse engineering. Finally, we demonstrate high biomass production in a fed-batch experiment and engineer lactate production from formate by an optimized *E. coli* strain.

## Materials and methods

### Chemicals and reagents

Primers were ordered from IDT (Leuven, Belgium). PCR reactions were performed using either Phusion High-Fidelity DNA Polymerase for gene amplification or DreamTaq DNA Polymerase for colony PCR (Thermo Fisher Scientific, Dreieich, Germany). Restriction digestions were carried out using FastDigest restriction enzymes and for ligation reactions T4 DNA ligase was used (all from Thermo Fisher Scientific). Sodium formate was ordered from Sigma-Aldrich (Steinheim, Germany).

### Bacterial strains

The formatotrophic *Escherichia coli* strain [based on *E. coli* MG1655 (F^−^ λ^−^ *ilv*G^−^ *rfb*-50 *rph*-1)] equipped with reductive glycine pathway (K4e) (Kim et al., 2020) was used as base strain for adaptive evolution and reverse engineering. *E. coli* DH5α (F^−^ λ^−^ Φ80*lac*ZΔM15 Δ(*lac*ZYA-*arg*F)U169 *deo*R, *rec*A1 *end*A1, *hsdR*17(rK^−^ mK^+^) *pho*A *sup*E44 *thi*-1 *gyr*A96 *rel*A1) was used for cloning. *E. coli* ST18 (*pro thi hsdR* ^+^ Tp^r^ Sm^r^; chromosome::RP4-2 Tc::Mu-Kan::Tn7λ*pir*Δ*hemA*) was used for conjugation. All strains are listed in Table 1.

**TABLE 1:** Strains and plasmids used in this study.

Strain/plasmid	Description/genotype	Source
*Strains*		
MG1655	F^−^ λ^−^ *ilv*G^−^ *rfb*-50 *rph*-1	Blattner et al. (1997)
DH5α	Laboratory cloning strain, F^−^ λ^−^ Φ80*lac*ZΔM15 Δ(*lac*ZYA-*arg*F)U169 *deo*R *rec*A1 *end*A1 *hsd*R17(rK^−^ mK^+^) *pho*A *sup*E44 *thi*-1 *gyr*A96 *rel*A1	Meselson and Yuan, (1968)
ST18	5-aminolevulinic acid auxotrophic strain for diparental mating, *pro thi hsdR* ^*+*^ Tp^r^ Sm^r^; chromosome::RP4-2 Tc::Mu-Kan::Tn7/λpir Δ*hem*A	Thoma and Schobert, (2009)
SerAux	MG1655 derivative, serine-auxotrophic strain, Δ*ser*A Δ*lta*E Δ*kbl* Δ*ace*A	Yishai et al. (2018)
K4, gC_1_M gC_2_M gC_3_M gEM	SerAux-based formatotrophic strain, gC_1_M gC_2_M gC_3_M, ss10-P_STRONG_-RBS_A_-*fdh*	Kim et al. (2020)
K4e	K4-based strain with ALE selected mutations in pntA*, fdh*	Kim et al. (2020)
K4e2, g-pdhR*, g-ackA*	K4e-based strain with ALE selected mutations in *pdhR* (E239X) and mobile element insertion in the promoter region of *ackA*	This study
K4eΔ*ackA-pta*	K4e with deletions in *ackA*-*pta*	This study
K4e2Δ*ackA-pta*	K4e2 with deletions in *ackA*-*pta*	This study
KS44	K4e with deletions in *ackA*-*pta* and *dld*	This study
KS46	KS44 overexpressing ldhA from pSStac-ldhA	This study
***Plasmids***		
pSStac	Overexpression plasmid with p15A origin, streptomycin resistance, tac promoter	This study
pSStac-*ldhA*	pSStac backbone for overexpression of *ldhA* from *E. coli*	This study

### Genome engineering

Genes were knocked out by P1 phage transduction (Thomason et al., 2007). Lysates for knockouts were generated from Keio collection strains (Baba et al., 2006). For selection marker removal (all flanked by FRT (flippase recognition target) sites), cells were transformed with a flippase recombinase plasmid (FLPe; temperature sensitive ORI, Gene Bridges, Heidelberg, Germany). Temperature increase to 37°C was used for flippase expression and to cure the cells from the FLPe plasmid.

### Synthetic-operon construction

A gene native to *E. coli*, lactate dehydrogenase (*ldhA*), was prepared *via* PCR amplification from the *E. coli* MG1655 genome. Using the previously described method (Zelcbuch et al., 2013), PCR product was cloned into pNiv, a high-copy-number cloning vector. Afterwards the gene was cloned into a pZ vector (15A origin of replication, streptomycin marker) by ligating the insert restricted with EcoRI and PstI from pNiv into similarly restricted pZ backbone. The promoters and RBS used were described previously (Braatsch et al., 2008; Zelcbuch et al., 2013).

## Growth medium and conditions

LB medium (1% NaCl, 0.5% yeast extract, 1% tryptone) was used for strain maintenance and molecular biology work. Growth experiments were carried out in M9 minimal media (50 mM Na_2_HPO_4_, 20 mM KH_2_PO_4_, 1 mM NaCl, 20 mM NH_4_Cl, 2 mM MgSO_4_, and 100 μM CaCl_2_) with trace elements (134 μM EDTA, 13 μM FeCl_3_·6 H_2_O, 6.2 μM ZnCl_2_, 0.76 μM CuCl_2_·2 H_2_O, 0.42 μM CoCl_2_·2 H_2_O, 1.62 μM H_3_BO_3_, 0.081 μM MnCl_2_·4 H_2_O). For growth experiments, LB overnight cultures were used to inoculate 4 mL M9 preculture containing 10 mM glucose, 1 mM glycine, and 30 mM formate in 10 mL glass tubes to an optical density (600 nm, OD_600_) of 0.02. All glass tube cultures were incubated at 37°C with shaking at 240 rpm. To inoculate growth experiments cells from precultures were centrifuged (18,407 × *g*, 3 min, 4°C), washed two times in M9 medium, and used to inoculate M9 containing the indicated carbon sources. Experiments were carried out in aerobic conditions either in 10 mL glass tubes or in 96-well microplates (Nunc, Thermo Fisher Scientific). In both conditions, an atmosphere of 90% air and 10% CO_2_ was supplied in incubator and platereader, respectively. When following growth in platereader experiments, microtiter plates contained 150 μL culture and (to avoid evaporation) the culture was covered with 50 μL mineral oil (Sigma-Aldrich). Growth experiments were conducted at 90% air/10% CO_2_ in BioTek Epoch 2 plate readers (Agilent, Santa Clara, CA, United States) at 37°C. OD_600_ was measured at the end of 12 shaking steps, that were alternating between linear and orbital (1 mm amplitude) shaking for 60 s. Plate reader measured OD_600_ values were converted to cuvette OD_600_ values according to OD_cuvette_ = OD_plate_/0.23. When experiments were carried out in glass-tubes, volume loss by evaporation was corrected by adding sterile water. Growth data shown represents averages of growth experiments performed in triplicate.

### Lactate production experiments

Colonies from LB plates were used to start test tube LB over-night precultures. Strains were washed 3 times with M9 medium. Each strain was then inoculated in test tubes with starting OD_600_ of ∼ 0.05. All tubes were incubated in an orbital shaker at 37°C with 10% CO_2_ until the stationary phase in each treatment was reached. Selected cultures were fed with formic acid when each culture reached the stationary phase (OD stopped increasing). Formic acid was added to each tube to increase its concentration by either 30 mM or 60 mM. For each strain, control tubes were left without feeding. Expression of *ldhA* was induced by adding 1 mM isopropyl β-d-1-thiogalactopyranoside (IPTG) together with additional formic acid after 120 h of cultivation. Periodic sampling was performed for measuring the extracellular ions dissolved in the medium by ion chromatography (Dionex ICS 6000 HPAEC, IonPac AS11-HC-4µm Analytical/Capillary Column; Thermo Fisher Scientific). At each sampling point, 200 µL were taken from the cultures and centrifuged at 15,000 rpm for 3 min. The supernatant was then diluted 20 times with ddH_2_O. The diluted sample was centrifuged again and transferred to a chromatography vial for the ion chromatography analysis.

### Dry-weight analysis

Biomass yield was determined by dry cell weight (CDW) measurement of triplicate cultures. *E. coli* cells were inoculated to a starting OD_600_ of 0.01 into M9 medium containing 90 mM of formate in 125 mL flasks. Cultures were incubated in the presence of 10% CO_2_ at 37°C with shaking at 240 rpm. Cells were harvested in exponential growth phase (OD_600_ of 0.6–0.8) on 90 mM formate. Samples of up to 50 mL were harvested by centrifugation (3,220 × *g*, 20 min) and washed three times in H_2_O to remove residual medium compounds (7,000 × *g*, 5 min). Finally, the cells were resuspended in 2 mL H_2_O, transferred into pre-dried aluminum dishes, and dried for 16 h at 90°C. CDW was determined and subtracted by the dish weight.

## Results and discussion

### Adaptive laboratory evolution leads to improved formatotrophic growth characteristics

We previously developed a formatotrophic *E. coli* strain named K4e (Kim et al., 2020). Engineering of this strain was achieved following a modular strategy that included four different modules: i) the C_1_ module for the conversion of formate into methylene-THF (*Methylobacterium extorquens* formate THF ligase, methenyl-THF cyclohydrolase, and methylene-THF dehydrogenase); ii) the C_2_ module condensing methylene-THF, CO_2_, and ammonia to glycine (endogenous enzymes of the glycine cleavage system; GCS, GcvT, GcvH, and GcvP); iii) the C_3_ module, condensing glycine and methylene-THF to serine, and finally deaminating serine to pyruvate (endogenous serine hydroxymethyltransferase (SHMT) and serine deaminase); and iv) the energy module generating NADH from formate [formate dehydrogenase (FDH) from *Pseudomonas* sp. (strain 101)] (Kim et al., 2020). After initial growth was observed, the strain’s growth was optimized, reaching performance characteristics of isolated mutants (K4e) of 9 h doubling time and a biomass yield of 2.3 g CDW/mol formate. Subsequent genome sequence analysis of K4e and reverse engineering revealed that upregulation mutations in the energy module and the membrane-bound transhydrogenase (a gene product of *pntAB*) supported enhanced growth on formate.

**FIGURE 2:**
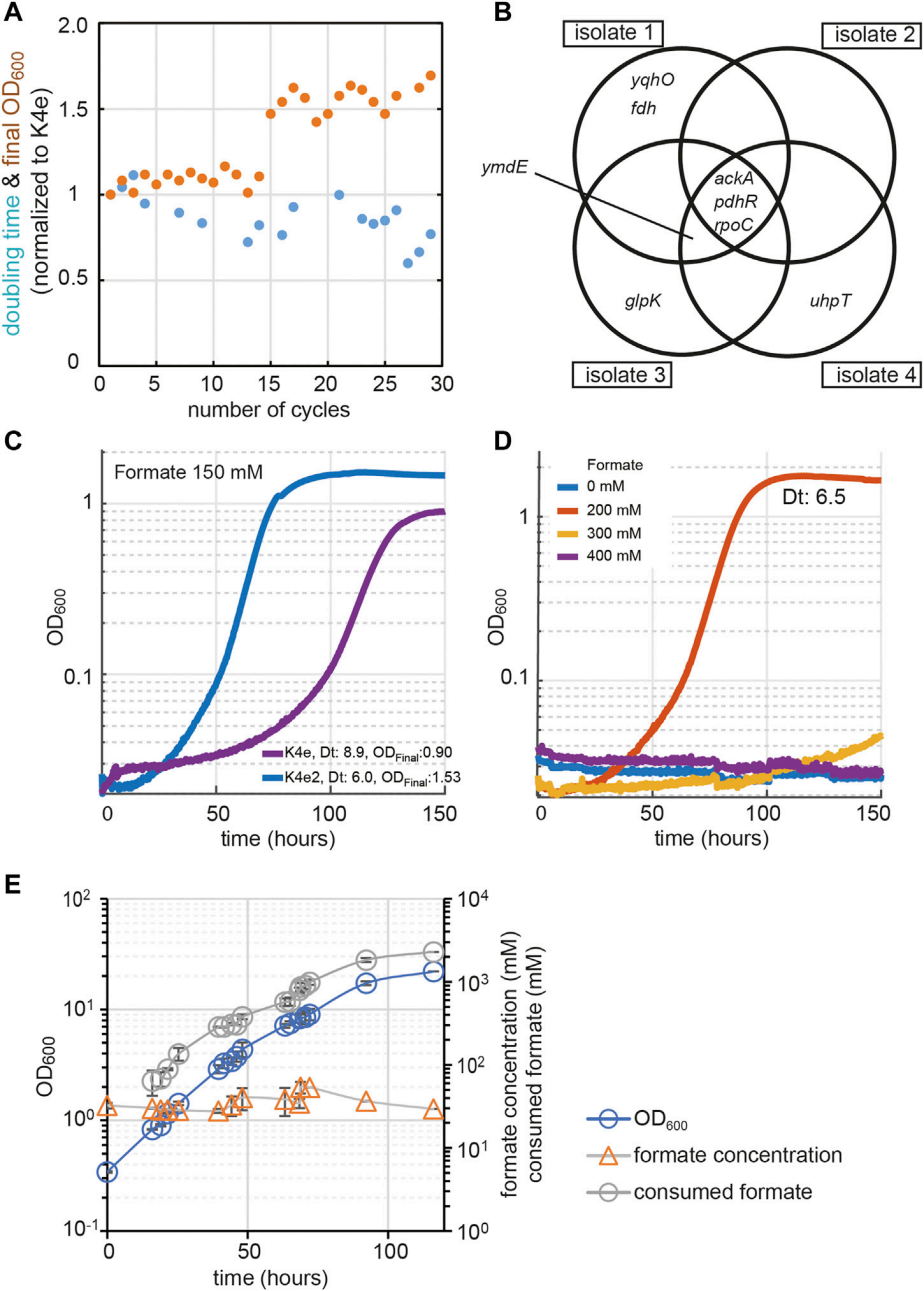
Evolution approaches for enhancing growth on formate. **(A)** Evolution from K4e to K4e2 *via* laboratory evolution was conducted in test tubes in M9 minimal medium with 90 mM formate in the presence of 10% CO_2_. Final OD_600_ (orange circle) and doubling times (blue circle) were normalized to K4e. **(B)** All identified mutated genes obtained after 30 cycles of re-inoculation. Only newly identified mutations are shown compared with parental strain K4e. **(C)** Growth profile comparison between evolved strains. **(D)** Formate tolerance test of K4e2. **(E)** Fed-batch cultivation of strain K4e2 in a bioreactor with pH control. Feeding and pH control were achieved by pumping 10 M of formic acid. List of genes: *pdhR*, pyruvate dehydrogenase complex regulator; *ymdE*, uncharacterized protein; *ackA*, acetate kinase; *yqhO*, biofilm formation related gene; *rpoC*, RNA polymerase subunit beta; *glpK*, glycerol kinase; Experiments **(C,D)** were carried out at 10% CO_2_ in 96-well plates in triplicates, displayed results are averages with errors < 5%. The corresponding doubling times (Dt) are shown in the figure.

To improve K4e′s growth performance further, we used this strain in an adaptive laboratory evolution (ALE) experiment, selecting for faster growth on formate and CO_2_. The cells were grown in M9 minimal medium containing formate and CO_2_ as the sole carbon sources. We cultivated the K4e strain in test tubes with a formate concentration of 90 mM in a CO_2_ atmosphere set to 10%. Once the turbidity reached an OD_600_ of 1.0 (corresponding to late exponential phase), the culture was diluted 1:100 into fresh medium of the same composition to start a new cultivation cycle. While the doubling time gradually decreased over 30 cycles, the final OD_600_ was stagnant for the first 14 cycles (≤90 generations). From cycle 15 onwards it appeared that a new mutant became dominant and a stairway-like enhancement in OD_600_ was observed (Figure 2A). To confirm the growth improvement of individuals from the ALE culture, growth of four independent isolates (originating from cycle 26) was analyzed in the plate reader. These independent growth tests conducted with the newly isolated strains, named as ‘K4e2’, confirmed > 40% faster growth of the isolates, reflected by a decrease in doubling time from 9 to 6.3 h. Strikingly, the isolates also showed a 40% increase in biomass yield, from 2.3 to 3.3 g-CDW/mol formate. Furthermore, increased tolerance toward formate was observed for the newly isolated strain K4e2, which showed accelerated growth as expressed by a reduced doubling time from 8.9 to 6 h and an increase of the final OD_600_ from 0.9 to 1.53 at 150 mM formate (Figure 2C). Moreover, even with 200 mM of formate, the strain grows without any significant growth-rate reduction (Figure 2D, 6.5 h doubling time). Compared to K4e, which showed optimal growth at < 90 mM formate and poor growth at > 150 mM (Kim et al., 2020), K4e2 represents a clear improvement. Thus, the isolated strain not only improved in biomass productivity, but also in formate tolerance, a feature, that is especially beneficial in terms of bioprocess design, since the system can be more robust with strains that exhibit higher formate tolerance.

To reveal the genetic changes underlying the growth improvements of the K4e2 isolates, the genomes of all four isolates were sequenced (see Supplementary Table S1 for all mutations identified). The analysis revealed that all four isolates share three common mutations. The first mutation is a mobile element insertion in the promoter region of *ackA*, which encodes an acetate kinase, responsible for acetate uptake or acetate overflow metabolism in the presence of oxygen (Wolfe, 2005; Szenk et al., 2017). The second mutation is a non-sense mutation (E239X) in *pdhR*, encoding a DNA-binding transcriptional dual regulator. The gene product of *pdhR* represses genes of the pyruvate dehydrogenase complex (PDH) and in the terminal electron transport systems (Ogasawara et al., 2007). As PDH converts pyruvate—a product of formate assimilation *via* the rGly pathway—to acetyl-CoA, a change in gene expression brought about by a *pdhR* mutation might positively influence growth by decreasing oxidative flux *via* the TCA cycle. Moreover, the enzyme complexes of PDH and GCS, the key enzyme of the rGly pathway, both contain lipoamide dehydrogenase, the expression of the corresponding gene (*lpd*) is repressed by PdhR (Quail and Guest, 1995). Lastly, a point mutation (A919V) occurs at *rpoC*, which encodes RNA polymerase subunit β’. Here, a direct relevance to carbon and energy metabolism is not obvious (Figure 2B).

### Blocking of acetate overflow metabolism improves formatotrophic growth

**FIGURE 3:**
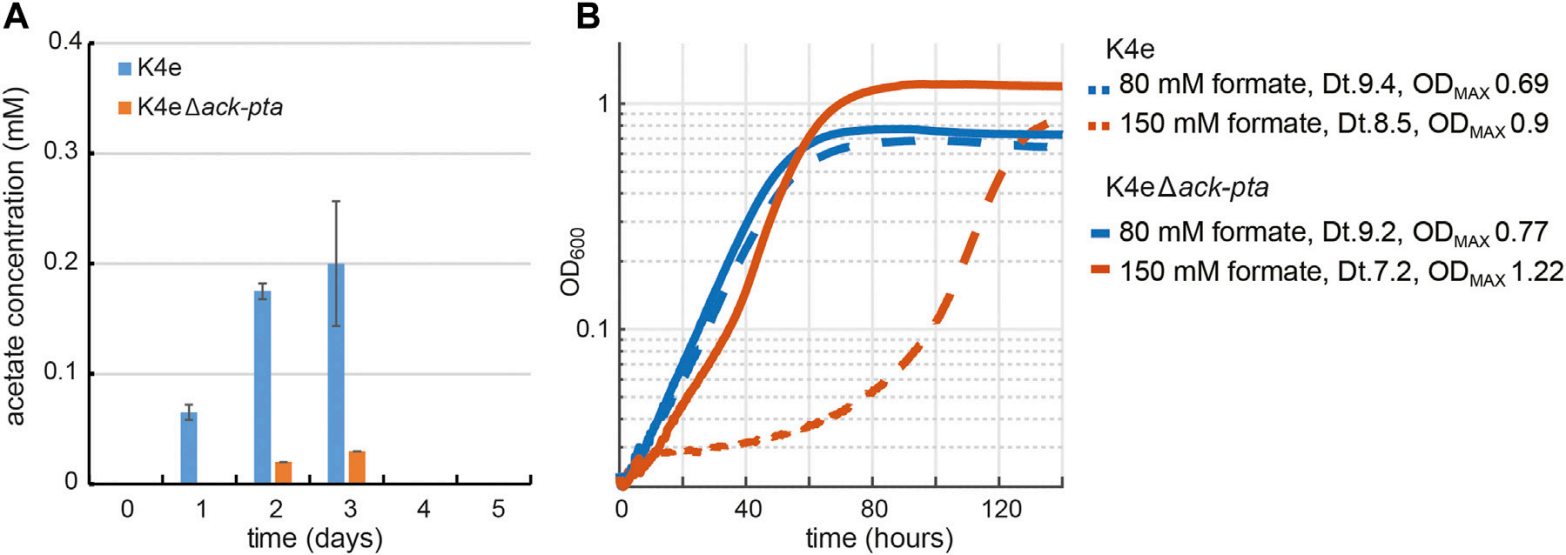
Effect of acetate overflow metabolism on the growth of K4e. **(A)** Acetate accumulation of K4e in test tubes with 90 mM initial formate. Only acetate was excreted in K4e and re-assimilated as the cell growth enters exponential phase. Strongly reduced acetate production was observed with K4e ∆*ackA-pta*. **(B)** Growth profile of K4e and K4e∆*ackA-pta* on 80 and 150 mM formate in 96-well plate experiments performed in triplicates, which displayed identical growth curves (<5% errors). Deletion of *ack-pta* resulted in increasing final OD_600_ and high formate tolerance. Dt, doubling time.

Among the mutations found in the K4e2 isolates from the evolution experiment, the mobile element (ME) insertion into the upstream region of *ackA* (acetate kinase) provides important information regarding the formatotrophic growth mode of *E. coli via* the rGly pathway. The ME insertion occurred at the −35 element in the promoter region of *ackA*, which we assume would decrease the level of *ackA* expression. It is not intuitive to consider the occurrence of acetate overflow metabolism in *E. coli* while growing on formate and reaching only very limited final OD_600_ (Basan et al., 2015; Bernal et al., 2016). However, the observed ME insertion suggests that by-product generation might be a limiting factor while growing on formate. Hence, we measured accumulation of metabolites, including acetate, succinate, lactate, and pyruvate during formatotrophic growth. Acetate accumulation was indeed observed from an early growth stage and reached up to 0.2 mM in K4e (Figure 3A). Besides acetate, no other organic acid was measurable in the analyzed samples. We found that the excreted acetate is re-assimilated as the cell enters the mid-exponential phase. This can be facilitated by either acetate kinase (*ackA*) and phosphotransacetylase (*pta*), consuming one ATP, or acetyl-CoA synthetase (*acs*), which converts acetate to acetyl-CoA while consuming two ATP equivalents (Kumari et al., 1995). In order to prevent K4e from synthesizing acetate, causing loss of carbon and to mimic the ME insertion found in the K4e2 isolates, both *ackA* and *pta* were deleted, resulting in the K4eΔ*ackA*-*pta* strain. When K4eΔ*ackA*-*pta* was cultured using the same conditions, acetate excretion was strongly reduced. To compare the growth performance of K4e and K4eΔ*ackA*-*pta*, we used two different formate concentrations. With 80 mM formate, both strains show similar growth patterns with almost identical doubling times. However, with 150 mM formate, the K4eΔ*ackA*-*pta* strain displays not only a reduced doubling time of 7.2 h (as compared to 8.5 h of K4e), but also exhibits a 30% increase in the final OD_600_ (Figure 3B). Indeed, we determined the observed biomass yield of K4eΔ*ackA*-*pta* to be 3.1 g CDW/mol formate, close to that of K4e2 (3.3 g CDW/mol formate). Moreover, the apparent lag phase of K4e on 150 mM formate was not present in K4eΔ*ackA*-*pta*, thus the K4eΔ*ackA*-*pta* strain grew within 80 h to the stationary phase, while the parental strain required more than 140 h to reach the stationary phase. As we expected, avoiding acetate excretion by deleting acetate biosynthesis in K4e2 led to increased biomass yield on formate. Thus, abolishing acetate biosynthesis in K4e is apparently helpful for growth on formate. The inactivity of acetate synthesis removes the option of ATP generation *via* acetate kinase, to compensate for this ATP generation option, an increased TCA cycle flux to generate additional NADH and ATP could be the consequence. Additionally, as the K4e strain carries a deletion in *aceA,* encoding isocitrate lyase, the strain lacks a functional glyoxylate shunt, which is essential for acetate assimilation as the sole carbon source. Thus, acetate, when assimilated into the TCA-cycle, mainly generates reducing power *via* the TCA-cycle and contributes only to a small fraction of some biomass building blocks (e.g. fatty acids, leucine, and glutamate-family amino acids). This might be one reason for the better growth of the *ackA-pta* deletion strain. A further explanation for the growth optimization is, that upon deletion of *ackA-pta* RNA polymerase sigma factor (RpoS) is upregulated. RpoS is a major regulator of the general stress response in *E. coli* and positively affects resistance to acid stress (Kirkpatrick et al., 2001). Since, the deletion of *ackA-pta* in the naïve strain already improved growth performance to the level of the K4e2 strain, reverse engineering of *rpoC* and *pdhR*, that cannot directly be connected to carbon and energy metabolism, was not pursued.

**FIGURE 4:**
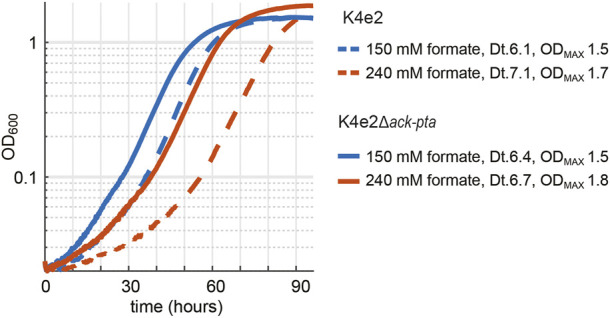
Deletion of *ack-pta* in K4e2 further optimizes formatotrophic growth. Experiments were carried out at 10% CO_2_ in 96-well plates in triplicates, displayed results are averages with errors < 5%.

To further investigate if a complete deletion of *ackA* and *pta* in the evolved K4e2 strain would positively influence the strain’s growth performance, we deleted both genes in the K4e2 strain, yielding K4e2Δ*ackA*-*pta.* A direct comparison of growth of K4e2 to K4e2Δ*ackA*-*pta* revealed no difference in terms of doubling times and final OD_600_ when strains grew with 150 or 240 mM formate (Figure 4). However, K4e2Δ*ackA*-*pta* clearly displays a reduced lag phase before the onset of exponential growth, suggesting that the complete deletions allow the strain to use the formate more efficiently. However, this is not reflected in the observed biomass yield of 3.4 g CDW/mol formate, which is virtually identical to the biomass yield of the parental strain K4e2. However, the biomass yields achieved by K4e2 exceeds the reported average biomass yields of microorganisms naturally growing on formate *via* the Calvin–Benson–Basham cycle (3.2 g CDW/mol formate) (Claassens et al., 2019).

### Fed-batch cultivation for high biomass generation

In order to characterize the strain’s potential for large-scale production we conducted fed-batch cultivation in a 1-L stirred-tank bioreactor, growing strain K4e2 in M9 medium with 30 mM formate at pH 7. For controlling pH and feeding of formate we used 10 M formic acid only. Starting from an inoculation OD_600_ of 0.34, the strain reached a final OD_600_ of 22 within 116 h (corresponding to six doublings) with a maximal growth rate of 0.048 h^−1^, corresponding to a doubling time of 14.4 h. The total biomass produced corresponded to 8 g CDW/l (Figure 2E). With a total consumption of 2.289 mol/L formate, the observed biomass yield was determined to be 3.5 g CDW/mol formate, which is consistent with the values derived from batch cultivations. The achieved cell density and growth velocity largely exceeds those previously reported for engineered formatotrophic *E. coli* (Bang et al., 2020). This highlights the potential of the engineered strain for use in industrial bioprocesses, where high cell densities are often required to achieve economic feasibility. However, some improvement with respect to biomass yield and doubling time is still possible, especially when comparing to the reported maximal theoretical biomass yields of ∼ 5 g CDW/mol formate (Bar-Even et al., 2013; Cotton et al., 2020). We thus set out to further improve our strains by analyzing and making use of mutations that accumulated during the adaptive evolution.

### Formatotrophic lactate production in *E. coli*

**FIGURE 5:**
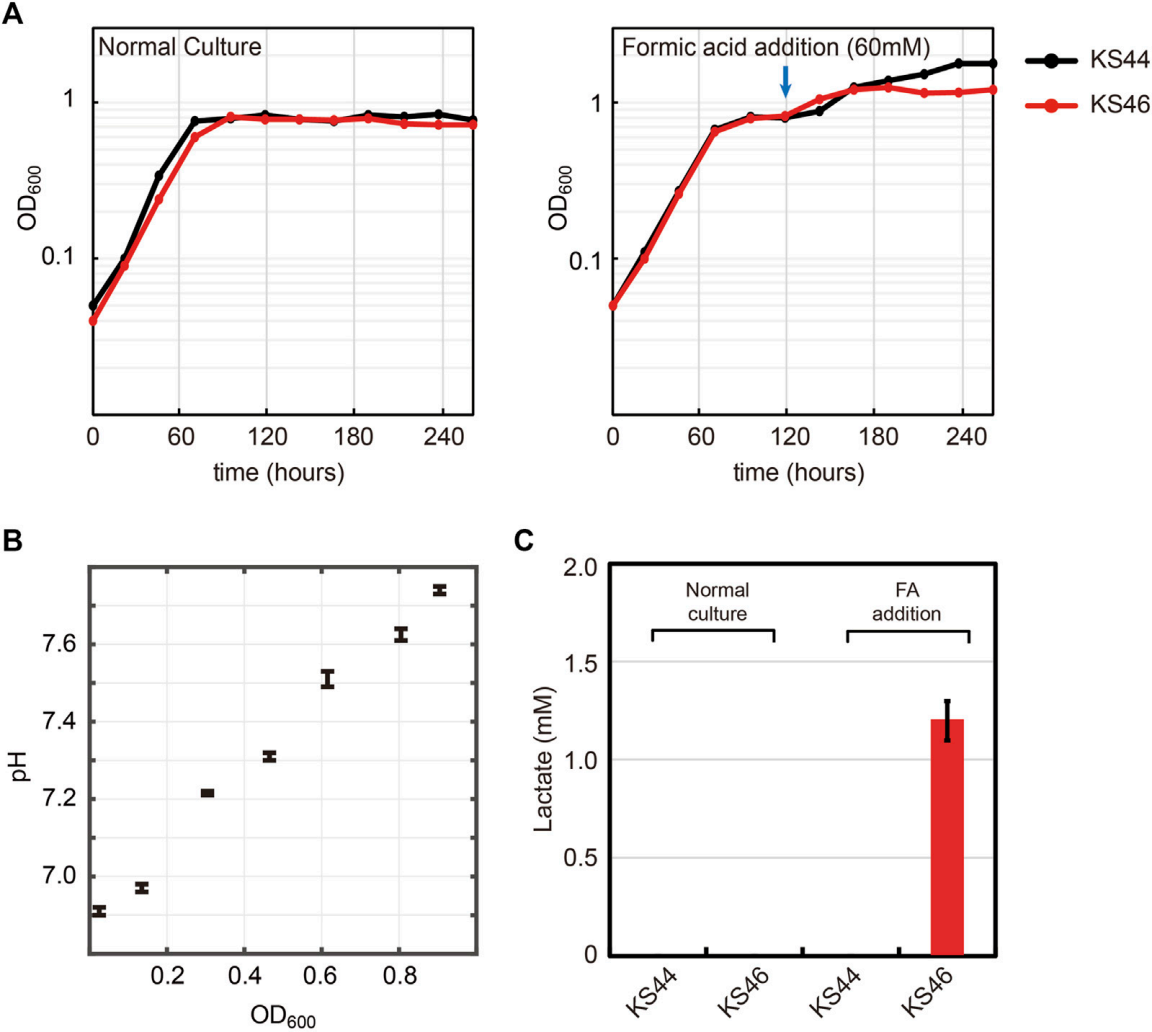
Lactate production from formate with formatotrophic *E. coli*. **(A)** Engineered *E. coli* strain was cultured in minimal medium using 90 mM formate and 10% CO_2_ as carbon sources. Two different cultivation methods were tested: normal batch mode and fed-batch mode with the addition of 60 mM formic acid (FA) at the indicated time point. **(B)** pH profile during growth on formate. Cell growth on formate directly correlates with increased medium pH due to the accumulation of OH^−^. **(C)** Lactate production was observed only with the strain in the fed-batch mode along with *ldhA* overexpression (n = 4, lactate measurement was conducted at the end of cultivation). Experiments were carried out at 10% CO_2_ in incubator shaker. Displayed results are averages with errors < 5%.

Lactate was selected as a proxy chemical to show the potential of formatotrophic bioproduction. Lactate is an important chemical used in the food and chemical industry. It contains a hydroxyl as well as a carboxyl group and can form poly-lactic acid by self-esterification, a polymer for producing bio-plastic (Maki-Arvela et al., 2014). Lactate can be generated by a reaction catalyzed by lactate dehydrogenase (*ldhA*), which oxidizes NADH using pyruvate as an electron acceptor. In order to prevent *E. coli* from re-assimilating the lactate, quinone dependent D-lactate dehydrogenase (*dld*) was deleted, generating K4eΔ*ackA*-*pta*Δ*dld*, named KS44. To achieve formatotrophic lactate production, the KS44 strain was transformed with the *ldhA* gene cloned into an IPTG-inducible expression cassette in plasmid pSStac. The final strain with *ldhA* overexpression was named KS46. To test for IPTG-inducible lactate production from formate, we applied a two-phased strategy. The growth phase was started in 90 mM formate and continued until the cells entered the stationary phase. The production phase was initiated by adding 1 mM IPTG and 60 mM of formic acid to the culture (Figure 5A). During the growth phase, the pH of the culture increases due to formate uptake into the cell, either *via* a proton symport mechanism or in the form of free formic acid (Wang et al., 2009; Lü et al., 2011; Wiechert and Beitz, 2017). Assimilation of 90 mM formate increased the culture pH from 6.9 to 7.8 (Figure 5B). Addition of 60 mM formic acid decreased the culture’s pH back from 7.8 to 6.9. Along with the two-phased strategy, a normal batch culture was also cultivated for comparison. When formate was completely consumed in the culture, lactate concentrations in the supernatant was analyzed. Here, we were able to detect 1.2 mM of lactate in the two-phase cultivation (Figure 5C), corresponding to almost 10% of the maximal theoretical yield (Cotton et al., 2020).

## Conclusion

This study demonstrates that a previously engineered formatotrophic *E. coli* strain growing *via* the rGly pathway can be optimized for the production of value-added chemicals such as lactate. Adaptive laboratory evolution conducted with CO_2_ and formate as carbon sources yielded a strain with increased biomass yield, shorter doubling time, and the ability to grow to high cellular densities. Interestingly, formate tolerance was increased as well, allowing growth with 200 mM formate, while the parent formatotrophic strain K4e did not grow at such formate concentrations. Subsequent genome sequencing revealed that avoidance of acetate production is one of the key factors for improved growth. By-product analysis of K4e showed that this strain indeed generates acetate during growth on formate and re-assimilates excreted acetate at the late stage of the growth phase. When acetate kinase and phosphate acetyltransferase were deleted from K4e, a similar growth-profile compared to K4e2 was observed, especially with high formate concentration. While the maximal cell density previously reported was 3.5 g CDW/l and the biomass yield 2.5 g CDW/mol formate (Bang et al., 2020), the newly evolved K4e2 strain exceeded those by reaching a cell density of 8 g CDW/l and a biomass yield of 3.4 g CDW/mol formate. This finding not only exemplifies the utility of adaptive laboratory evolution but also constitutes a further step towards the industrial use of the synthetic rGly pathway. This sustainable approach to bacterial biomass production can directly find application in areas like single-cell protein or feed production. However, even more urgent but more challenging is the development of sustainable processes for value-added chemicals to provide alternatives to petroleum-based sources.

In order to achieve biological transformation of formate to lactate, inducible lactate dehydrogenase was implemented and the lactate assimilating quinone-dependent D-lactate dehydrogenase was deleted. When formate/formic-acid fed-batch cultivation was carried out with this strain, production of lactate was observed. We thus showed that our formatotrophic *E. coli* strain, which utilizes formate as energy and carbon source through the rGly pathway, can be further optimized, in this case by prevention of the wasteful acetate formation, and can be applied for the microbial conversion to a chemical of interest. Finally, further strain engineering to increase flux towards lactate and the establishment of an optimized bioprocess will unlock the full potential of the rGly pathway and hence help paving the way towards a C1-bioeconomy.

In the presented study, we used ALE experiments to achieve faster growth *via* the rGly pathway. However, some additions to the genomic preset might be helpful to further optimize pathway efficiency and hence growth performance. E.g., changing the cofactor of the GCS to NADPH can provide a thermodynamic push, which might allow growth at lower CO_2_ concentrations. Additionally, improved ATP efficiency of the rGly pathway can increase growth efficiency. One way to achieve this is to replace the iron-sulfur-cluster enzyme serine deaminase, which provides limiting kinetic properties, with a transaminase converting serine to hydroxypyruvate which is entering glycolysis at the level of 3-phosphoglycerate.

## Data Availability

The original contributions presented in the study are included in the article/Supplementary Material, further inquiries can be directed to the corresponding author.
