# Supplementary material for: Optimizing E. coli as a formatotrophic platform for bioproduction via the reductive glycine pathway
Source: Front Bioeng Biotechnol. 2023 Jan 16;11:1091899. doi: 10.3389/fbioe.2023.1091899 (PMC9885119; doi:10.3389/fbioe.2023.1091899)
Supplement: Supplementary file 1 [file Table1.DOCX]

Supplementary Table 1. All mutations in K4e2 isolates compare to K4e

| **Mutation** | **Type** | **Annotation** | **Affected enzyme (gene)** |
| --- | --- | --- | --- |
| Point mutation | CDS | E239X (G🡪T) | pyruvate dehydrogenase complex regulator (*pdhR*) |
| Point mutation | CDS | A919V (C🡪T) | RNA polymerase subunit beta (*rpoC*) |
| Mobile element integration | UTR | - | acetate kinase (*ackA*) |
| polymerphism | CDS | - | biofilm formation related gene (*yghO*) |
| Point mutation | UTR | T 🡪 A | *Pseudomonas sp.* formate dehydrogenase (*fdh*) |
| Polymerphism | CDS | - | uncharacterized protein (*ymdE*) |
| Point mutation | CDS | V103I (G🡪A) | glycerol kinase (*glpK*) |
| Point mutation | CDS | Q96X (C🡪T) | hexose-6-phosphate:phosphate antiporter (*uhpT*) |
